# Anxiety and depressive symptoms among medical students—A scoping review of systematic reviews and meta-analyses

**DOI:** 10.3389/fpubh.2025.1710333

**Published:** 2026-01-02

**Authors:** Nadine Agyapong-Opoku, Felix Agyapong-Opoku, Belinda Agyapong, Andrew J. Greenshaw

**Affiliations:** 1 College of Health Sciences, University of Ghana Medical School, Accra, Ghana; 2 Department of Psychiatry, University of Alberta, Edmonton, AB, Canada; 3 School of Medicine, University of Galway, Galway, Ireland

**Keywords:** anxiety, depression, medical students, mental health, scoping review

## Abstract

**Background:**

Medical schools are globally recognized as higher education institutions requiring extreme dedication from students. The intensive nature of physician training demands heavy workloads, inconsistent sleep, and study-leisure imbalances. Such stressors are linked to poor student mental health, with anxiety and depression symptoms among the most documented disorders. These burdens negatively affect academic performance and are associated with dropout intentions, misconduct, burnout, and suicidal ideation.

**Objective:**

This scoping review summarizes recent evidence on the prevalence of anxiety and depression symptoms among medical students and identifies correlated factors.

**Methods:**

The review followed PRISMA guidelines and Arksey and O'Malley's five-stage methodological framework. Searches were conducted on July 5, 2025, in PubMed, MEDLINE, Web of Science, Scopus, and PsycINFO. Boolean operators combined terms related to prevalence, and correlates of depressive and anxiety symptoms, and medical students, limited to systematic reviews and meta-analyses published in English between January 2021 and July 2025. Sixteen studies met the inclusion criteria after screening. Data were charted for study characteristics, prevalence estimates, contributing factors, and methodological approaches.

**Results:**

The studies included in this review reported wide-ranging prevalence estimates, with the prevalence of depression symptoms in the included meta-analysis ranging from lowest of 18.1% to highest of 50.0% and anxiety symptoms from 17 to 54% although there was high heterogeneity in the screening instruments or measurement scales Biological sex differences in prevalence were frequently noted, with most studies reporting a higher prevalence among females; however, findings varied by region. Regional disparities were additionally observed, with some continents and countries reporting significantly higher prevalence rates than others. Factors associated with increased risk included early years of study, poor sleep quality, and academic stress. During COVID-19, most studies reported a higher prevalence of depression and anxiety symptoms than pre-pandemic levels.

**Conclusions:**

Anxiety and depressive symptoms remain widespread among medical students, driven by individual and contextual factors. Targeted interventions and early preventive strategies are urgently needed to address mental health challenges and protect student wellbeing.

## 1 Introduction

Medical schools are recognized globally as higher education institutions that require extreme professionalism and dedication from students (1). Students endure sleepless nights, extensive work hours, and copious amounts of learning material leading to an increased risk of psychological issues (2, 3). Many medical students report feeling guilty when not using their limited free time to study (4). Studies suggest a direct link between academic-related stressors in medical school and mental health decline (5, 6).

Among mental health disorders common among medical students, anxiety and depression are notable (7, 8). Multiple studies have shown that medical students experience higher rates of depression and anxiety than their age-matched peers and the general population (8–10). A 2016 meta-analysis reported a global prevalence among medical students of 33.8% for anxiety (9), and 28% for depression (11). These disorders influence dropout intentions (12) and academic performance (13–15). Other studies suggest these disorders increase the likelihood of academic misconduct (16), clinical dishonesty (17), burnout (18) and even suicide (19).

Given these concerning trends, researchers have investigated factors contributing to symptoms of anxiety and depression among medical students. Identified factors include biological sex, academic stress, low physical activity, low social support, poor sleep, lack of academic support, and year of study (20–22). Nevertheless, there are discrepancies across studies regarding the extent to which these factors contribute to the incidence of anxiety and depression symptoms among medical students. Studies have portrayed heterogeneous findings regarding possible causal effects of certain factors on this population, as conclusions vary across studies conducted in medical schools across the globe. Despite these inconsistencies, studies collectively reinforce an existing, and prominent presence of anxiety and depression symptoms among medical students.

This scoping review summarizes recent evidence on the prevalence of anxiety and depression symptoms among medical students, identifies contributing factors, and highlights gaps in the existing literature. It is essential to build on current knowledge of this topic to consider and determine appropriate interventions to support the mental health of medical students across the globe. Thus, highlighting the prevalence and correlates of anxiety and depression symptoms among medical students is vital for placing emphasis on the extent of the problem, encouraging medical schools to incorporate the fundamentals of mental wellbeing and health promotion throughout medical education. Such institutional action may also reduce or prevent subsequent burnout and the escalation of mental health disorders (23), which may have latent detrimental effects on medical students themselves, or the quality of care they will eventually provide to patients.

## 2 Methodology

### 2.1 Study design

The design for this review of reviews aligns with Arksey and O'Malley's five-stage approach to scoping reviews (24): (1) develop the research question; (2) identify relevant studies; (3) select articles; (4) chart the data; and (5) summarize and report the results.

### 2.2 Identifying the research questions

This scoping review's objective is to examine the scope of what is known about the prevalence and correlates of anxiety and depression symptoms among medical students.

### 2.3 Identifying relevant studies

On 5 July 2025, a systematic literature search was conducted using several electronic bibliographic databases, namely: PubMed (Public/Publisher MEDLINE (NLM journal articles database)), MEDLINE (Medical Literature Analysis and Retrieval System Online), Web of Science, Scopus Elsevier, and APA Psych Info. The search was conducted with the use of Boolean operators “OR”/“AND” between search terms. The specific search terms used were (Prevalence OR incidence OR epidemiology OR frequency OR occurrence OR Correlates OR associated factors OR predictors OR risk factors OR determinants OR associations) AND (medical students OR students in medicine) AND (depression OR depressive disorder OR depressive symptoms OR anxiety disorder OR anxiety symptoms) AND (systematic review OR Meta-analysis). The inclusion criteria were limited to systematic reviews, and meta-analyses written in English, and with emphasis on depression and anxiety symptoms among medical students. Gray literature, dissertations, preprints, conference proceedings, and all other review types were among the excluded articles. Publication restrictions of 5 years were applied (January 2021–July 2025) to ensure that the most up-to-date review literature was utilized during the period of data extraction.

### 2.4 Article selection

Articles were retrieved from selected databases and imported into Covidence, a web-based tool designed to support the screening of articles for systematic reviews (25). Duplicates were automatically removed. Two researchers (N.A.O and F.A.O) independently reviewed the citations during the title/abstract screening and the full-text review phase based on a specific eligibility criterion. All discrepancies were resolved through discussion and consensus to reach a majority vote decision and when necessary, a third reviewer (B.A) resolved conflicts.

Studies were only included if (1) medical students were the subjects of the study, (2) the prevalence and/or correlates of anxiety or depression symptoms were measured among the study population, and (3) studies were published between January 2021 and July 2025. Articles were additionally limited to systematic reviews and meta-analyses, written in English. Articles were excluded from the review if the study population of the review did not include medical students, and if the study population included medical students and other student populations but did not differentiate between these two groups in the presentation or interpretation of the results or discussion.

**Figure 1:**
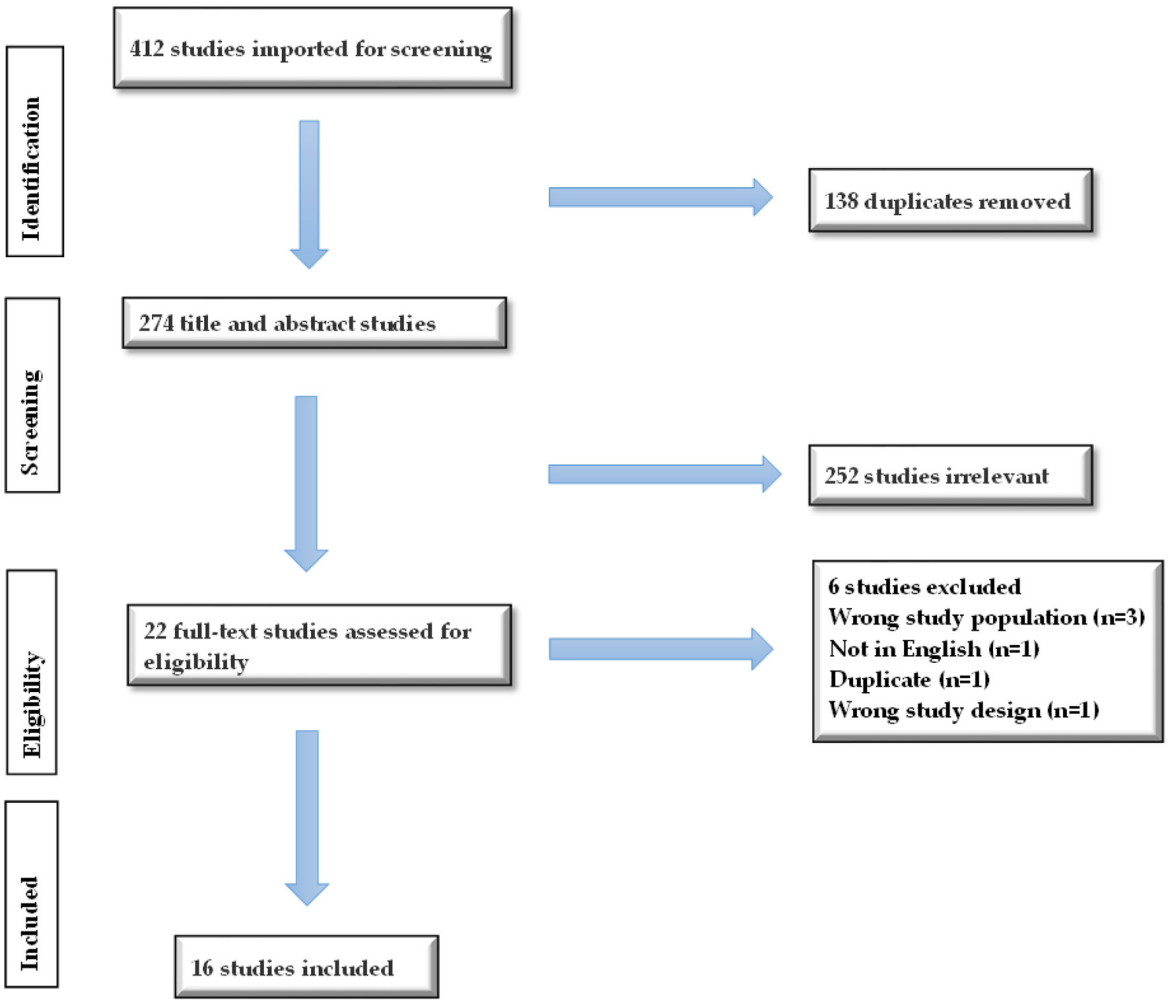
PRISMA diagram detailing the study identification and selection process in accordance with the Preferred Reporting Items for Systematic Reviews and Meta-Analyses (PRISMA).

We identified 22 articles for full-text review but excluded six articles that did not meet the inclusion criteria. Thus, 16 studies were included in the review as shown in Figure 1 below.

### 2.5 Data charting and extraction

The following information were extracted from the included articles according to the following domains: author(s) name, year of publication, total number of articles, review type, aim of review, participants, sample size (*N*), bias assessment, key findings (prevalence and correlates), and author recommendations.

### 2.6 Collating, summarizing, and reporting the results

This scoping review summarizes recent evidence regarding the mental health implications, specifically anxiety and depression symptoms, on medical student populations. Relevant data were summarized into tables and validated by two team members. The characteristics and results extracted from each article are summarized below.

## 3 Results

### 3.1 Overview of included articles

A total of 412 articles were identified from PubMed (36), MEDLINE (35), Web of Science (142), Scopus Elsevier (182), and APA PsycINFO (17). Covidence automatically removed 138 duplicates; 274 were left for title and abstract screening. Twenty-two studies qualified for full-text screening. After excluding six studies that did not meet the eligibility criteria, 16 studies were included in this review, as demonstrated in Figure 1. In total, 16 studies were included in this review. Eleven studies were classified as “Systematic Review and Meta Analysis,” three studies were “Systematic Review,” and two were “Meta-Analysis.” The included articles were published between January 2021 and July 2025. Of the included reviews, seven focused solely on depression symptoms among medical students, one focused solely on anxiety symptoms, and the remaining eight studies analyzed both anxiety and depression symptoms among medical students.

Of the included studies, *N* = 8 studies were conducted in specific countries. Specifically, *N* = 1 study was conducted in Saudi Arabia, *N* = 3 studies were conducted in India, *N* = 1 study was conducted in Pakistan, *N* = 2 studies were conducted in China, and *N* = 1 study was conducted in Africa. The remaining studies (*N* = 8) included several articles originating from a diverse range of countries and continents. This is demonstrated in Table 1.

**Table 1:** Prevalence and correlates of anxiety and depression among medical students.

**References**	**Time period**	**Number of included Articles; Review Type**	**Continent of study population**	**Bias assessment**	**Participant characteristics and sample size**	**Correlates**	**Prevalence**	**Recommendations**
AlJaber (38)	January 2010 and March 2019	18; Systematic Review	Saudi Arabia	Not assessed	8,839 medical students	Smoking is strongly associated with depression severity First year students are most susceptible to developing depressive symptoms Females are at a high risk of depression	The prevalence of depression ranged from 30.9 to 77.6% Mean prevalence of depression was 51.46%	Lectures should be administered to raise awareness concerning depression among medical students. Screenings should be conducted, and students should be screened by family physicians. Mentorship programmes to assist medical students diagnose d with depression
Ayubi et al. (34)	December 2019 to September 18, 2021	52; meta-analysis 108; systematic review	The most articles were from China (24%), India (10.2%) and Pakistan (7.4%), respectively and one study was a national-wide study	This study considered a possibility of selection bias as many of the included studies used online surveys and non- random sampling	NR	Not a primary focus	The pooled prevalence of anxiety was 44% and 49% for depression The prevalence of the mental states was higher among medical students in the Eastern Mediterranean Region than other WHO regions	Greater emphasis should be placed on screening, early diagnosis, and milder symptoms prior to the onset of more severe symptoms Application of preventative programmes
Azim et al. (39)	2004–2019	30; Systematic Review	Pakistan		NR	Study year Financial Difficulties Academic burden	Prevalence of depression ranged from 20 to 90%	Implementation of awareness programmes concerning anxiety and depression. Timely support should be provided to students to ensure secondary prevention. This study also suggested longitudinal models on communication skills and stress management. Peer mentorship programmes and mental healthcare systems were also suggested
Dutta et al. (27)	2019–2022	19; Systematic Review and Meta Analysis	India	Doi plot confirmed the absence of publication bias	5,944 medical students	Not a primary focus	The pooled prevalence of depression was 50.0% The pooled prevalence of depression among females was 38.0% The pooled prevalence of depression among males-34.0%	Implement regular screening and counseling services for depression Raise awareness among parents and medical educators regarding depression and depressive symptoms
Dwivedi et al. (30)	2014–2018	28; Meta Analysis	India	Publication bias was present	7,046 medical students	Not a primary focus	The pooled prevalence of depression was 40% Females had a slightly higher prevalence of depression than males	Stress management interventions should be implemented
Jia et al. (33)	January 2020 to February 2021 COVID-19	41; Systematic Review 31; Meta Analysis	-	According to Egger's regression test, there was no publication bias	36,608 medical students	Not a primary focus	The pooled prevalence of depression was 37.9% Pooled prevalence of anxiety- 33.7% Prevalence among medical students was higher than that of the general population and healthcare workers The pooled prevalence of anxiety and depression among Chinese medical students was 28.7 and 24.3% respectively The study additionally conducted subgroup analyses that indicated a variation in prevalence based on continent of study: Depression Europe-52.3% Asia-36.2% Anxiety Europe-23.2% South America- 49.0%	Further exploration should be done on the differences and influencing factors of mental health among medical students with different cultural backgrounds
Jin et al. (31)	(PubMed, from April 2000; Embase, from 1974; the Cochrane Database of Systematic Reviews, from July 1997; CNKI, from June 1999; Wanfang, from 1998) to April 2021	21; Systematic review and meta-analysis	China- North China and South China	No obvious publication bias was found in Begg's and Egger's tests	39,780 medical students	Medical students with sleep disorders were over 3 times more likely to report depression	27% pooled prevalence of depression in Chinese medical students Pooled prevalence: Male- 29.0% Female-26.0% Pooled prevalence differed in individuals who suffered from sleeping disorders compared to those who did not Sleep disorders prevalence- 41.0%. No sleep disorder- 18.0% Pooled Prevalence differed based on region: North China-34% South China-18%	Implementation of regular screening and appropriate interventions are recommended
Kaur et al. (29)	August 2015 to June 2023	^*^52; Systematic Review and Meta Analysis	India	DOI plot was used to confirm the absence of bias	15,557	Not a primary focus	Pooled prevalence: 48%- depression 54%- anxiety More females than males were affected from depression and anxiety	Provide counseling services, as well as long term policies and programmes. Regular mental health evaluations and support measures were also recommended
Lasheras et al. (37)	2020	8; systematic review with meta-analysis	89%- China United Arab Emirates, Iran, Brazil, India.	The risk of bias scores ranged from 6 to 9 out of a possible total of 9. Results from Egger's test imply that there was no statistically significant systematic relationship between the results of each study and its size	−11,710	There are several COVID-19 specific stressors implicated in mental health outcomes	28% pooled prevalence of anxiety The overall level of anxiety in medicine did not appear to be increased during the COVID-19 outbreak	To have uninterrupted provision of medical education, schools and institutions should ensure adequate learning environments
Lin et al. (35)	December 1, 2019, to March 15, 2023	130 systematic review and meta-analysis	Asia, North and South America, Middle East, Africa, Europe, Middle East	Begg's test and Egger's tests showed significant publication bias for depression. For anxiety, Begg's test showed no bias, but Egger's test showed significant publication bias	132,068 medical students	Pandemic related psychological impacts Continent was significantly associated with the pooled prevalence of anxiety and depression There was a higher prevalence of anxiety and depression in female medical students than male. This was also seen more in pre-clinical students vs. clinical year students. But they were not significant associations	The pooled prevalence of mental health outcomes for anxiety and depression was 45 and 48%, respectively Medical students in Asia were found to have a lower prevalence of anxiety and depression than in other regions Students in Africa, North America and South America had the highest prevalence of anxiety	Implementation of appropriate strategies to meet the phycological demands of this population and to protect their mental health
Mekonnen et al. (36)	January 2013 to May 2023	31; systematic review and meta-analysis	Africa	The absence of publication bias revealed by the egger's test and funnel plot	34,189 medical students	Females were 75% less likely to develop depression Being a second-year medical student resulted in a 74% decreased likelihood of developing depression Being male resulted in an increased likelihood of developing depression	38.80% pooled prevalence of depression among medical students in Africa Subgroup analyses were additionally conducted which discovered that the prevalence of depression varied based on the country 48.47%- Cameroon 65.86%- Sudan 18.76%- Nigeria 19.16%- Uganda	Early detection and prevention programs
Paz et al. (57)	December 2019 and July 2021	47; systematic review	27 countries across the globe Nine studies in China, one study in Australia, one Kazakhstan, three studies in Turkey, one study in Saudi Arabia, two studies in Germany, two studies in Japan, five studies in the United States of America, one study in Italy, one study in Nepal, one study in Libya, two studies in Canada, one study in Bangladesh, one study in Jordan, one study in Ireland, six studies in India, one study in Lebanon, two studies in Brazil, one study in Morocco, one study in Singapore, one study in Thailand, one study in Peru, one study in Pakistan, and one study in Russia	Inherent bias exists because of survey studies	Population was not pooled	COVID-19 impact- resulted in higher levels of depression and anxiety	Prevalence of both anxiety and depression differed from country to country	Implementation of appropriate support and research on interventions that could prevent risks of mental health decline
Peng et al. (22)	01 January 2020 to 01 April 2022	201; systematic review and meta-analysis	Most of the studies were from Asia (72, 35.8%) and East Asia (72, 35.8%), followed by Europe (24, 11.9%), South America (11, 5.4%), North America (10, 5%), and Africa (9, 4.4%). Only one study was carried out in Australia and two studies recruited participants from diverse geographical regions	Few studies provided a reasonable response rate, suggesting potential selection bias. Publication bias was also present for anxiety according to Egger's test	198,000 Medical students	Major correlates were not specific to anxiety and/or depression	The pooled prevalence of depression was 41%, and anxiety was 38%	Targeted interventions must be implemented. Identification of high-risk students
Santabárbara et al. (32)	December 1, 2019, to December 27, 2020	11; systematic review and meta-analysis	Asia (*n* = 8), but we also found studies from Africa (*n* = 2) and South America (*n* = 1)	Funnel plot and Egger's test confirmed absence of publication bias	The sample size ranged from 217 to 2,430 participants	Lower prevalence of depression was found for studies which took place in Asia (25%) as opposed to studies which took place in other continents (51%)	The estimated pooled prevalence of depression in medical students was 31% Age and biological sex did not display differences in prevalence	–
Wang et al. (62)	January 2000 to December 2020	197; systematic review and meta-analysis	China	Publication bias was found in the pooled prevalence analysis using Egger's test	294,408 medical students in China	Not a major focus	The overall pooled crude prevalence for depression was 29% and 17% for anxiety Anxiety and depression among medical students were much higher than in the general population	Timely screening and appropriate interventions must be conducted. More attention more additionally be paid to medical students with signs and symptoms of depression
Zatt et al. (26)	Studies up to 13 March 2018	34; met analysis	Three from Africa, nine from Asia, eight from Europe, two from Oceania, seven from South America, and five from the United States	Minimal bias was discovered	18,030 medical students	Being female, older, and in earlier years of study	The pooled prevalence of depression was 18.1%	–

NR, Not Reported.

### 3.2 Targeted conditions and key findings

The scales used to assess symptoms of anxiety and depression in the included manuscripts, though standardized, are not meant to be diagnostic. Studies indicated elevated levels of anxiety and depression symptoms among medical students, which is a growing concern due to its potential impact on students' long-term wellbeing. Of the 13 meta-analyses included in the review, the lowest recorded prevalence for depression was 18.1% (26) and the highest was 50.0% (27). Among the included meta-analyses, the pooled prevalence of anxiety symptoms ranged from 17% (28) to 54% (29). It is important to note that while this review included both meta-analyses and systematic reviews, the systematic reviews reported ranges of prevalence rather than pooled estimates, which limited comparability; these ranges were therefore noted but not included in this quantitative summary. Seven studies indicated varying degrees, of disparity with regards to the prevalence of depression symptoms among males and females. Most studies concluded that the prevalence of depression symptoms among female medical students was, to varying degrees, higher than that of males (27, 29–31); however, another review detailed no differences in prevalence regarding biological sex, or even age of the medical student (32). A systematic review and meta-analysis conducted in Africa, on the other hand, reported that females were 75% less likely than males to develop depression. There was a gap in the prevalence regarding the three studies conducted in India, where the lowest prevalence was 40% (30) and the highest was 50% (27) for symptoms depression. Four studies noted several disparities in the prevalence of both anxiety and depression symptoms between different regions and continents (31, 33–36). One study conducted in China determined a 34% prevalence of depression in medical students in North China, and an 18% prevalence of depression in medical students in South China (31). Another study noted that the prevalence of mental states was elevated among medical students in the Eastern Mediterranean Region than in other WHO regions (34). Similarly, a study reported lower prevalence of anxiety and depression symptoms among medical students in Asia and higher prevalence in Africa, North America, and South America (35).

Of the six studies that exclusively focused on depression and anxiety symptoms among medical students during the COVID-19 pandemic (31, 32, 34, 35, 37), the lowest recorded pooled prevalence for anxiety symptoms was 28% (37) and the highest was 45% (35). The prevalence of depression symptoms, on the other hand ranged from 31% (32) to 49% (34). Two articles reported pooled prevalences from studies in 2020 onwards with slight differences in prevalence of ±4 for anxiety and depression symptoms reported (22, 33). There was overlap in years for individual included studies in the meta-analysis and systematic review for effective demarcation of pre pandemic studies and post pandemic studies prevalence of anxiety and depression symptoms.

Various factors were identified as contributing to the decline in mental health among medical students. Three studies indicated an increased susceptibility of depressive symptoms in students in their preliminary years of study as opposed to subsequent years of study (26, 38, 39). One study conducted in Africa reported that second-year medical students had a 76% decreased likelihood of developing depression compared to their counterparts (36). Another study observed that year of study was not significantly associated with depression or anxiety symptoms; however, the same study observed a higher prevalence of depression and anxiety symptoms in preclinical students than clinical students (35). A single study found a strong association between smoking and depression severity in medical students (38), whereas another study noted that medical students battling sleep disorders were over three times more likely to report depression, compared to medical students without sleep disorders (31). The biological sex of medical students yielded heterogenous discussion between studies in terms of its association with depression and anxiety symptoms. Three studies concluded that females were, to various degrees, at a higher risk of depression than males (26, 30, 38); however, another study conducted among African medical students refuted this idea and reported that females were 75% less likely to develop depression than males (36). Another study found an increase in the prevalence of depression and anxiety symptoms in female medical students but determined that this association was not significant (35). On the other hand, this study found continent of study to be significantly associated with the pooled prevalence of depression and anxiety symptoms.

## 4 Discussion

### 4.1 Overview

This scoping review investigated reports of the prevalence and correlates of anxiety and depression symptoms among medical students throughout their medical school studies. The findings were multidimensional and heterogeneous, highlighting the need for further research employing standardized assessment tools to improve data comparability. Variations in results may be attributed to variations in study designs, including but not limited to sample size, regional contexts, diversity in the standardized scales used for the assessment, regional contexts, as well as period of data collection. Despite these inconsistencies, several distinct trends emerged and are discussed below.

### 4.2 The impact of COVID-19 on student mental health

Of the six meta-analyses within this data set that specifically focused on depression and/or anxiety symptoms of medical students during the COVID-19 pandemic, only one directly compared pre-pandemic and pandemic-era anxiety levels (37). The study found no significant increase in anxiety symptoms among medical students during the pandemic, in contrast to a rise observed among non-medical students and the general population. The study attributed this difference to medical students' greater knowledge of COVID-19 treatment, prognosis, transmission, and prevention, which was found to be negatively correlated with anxiety levels. Additional studies determined that lower levels of education were associated with a higher risk of depression (40, 41). This is supportive of the fact that the higher educational attainment of medical students may have provided some resilience against pandemic related mental health challenges. Other studies have found that a higher knowledge base regarding the disease serves as a positive factor that mitigates mental distress levels (42, 43). Additionally, these findings may be understood through the lens of the Cognitive Appraisal Theory, which suggests that stress responses are determined by an individual's coping capacity and evaluation of threat (44). Medical students' increased knowledge of disease transmission and treatment may have led to less threatening evaluations of the disease, thus mitigating anxiety levels during the pandemic, as the study suggests.

Nonetheless, pre-pandemic baseline data reveal a concerning trend. Tian-Ci Quek et al. (9) reported a global anxiety prevalence of 33.8% among medical students before the pandemic. Among the five meta-analyses in this review assessing pandemic-era anxiety, three reported rates exceeding this figure by more than 5%, while two reported lower prevalence rates. This variability may reflect differences in study populations, measurement tools, or regional factors. Similarly, Puthran et al. (11) estimated a global depression prevalence of 28% among medical students prior to the pandemic, while all five pandemic-era meta-analyses in this review reported higher rates. The contrast between the listed studies may be suggestive of the fact that, despite some protective factors linked to the education attainment of medical students, COVID-19 inevitably exacerbated their mental health burden to variable extents.

A growing body of evidence supports this conclusion. COVID-19-specific stressors, including social isolation, academic disruption, and misinformation, were strongly associated with deteriorating mental health (40, 45). Globally enforced quarantine and self-isolation measures, while necessary for infection control, were associated with increased feelings of loneliness, dejection, self-harm, insomnia, and risky substance abuse (46). Circulation of rumors and misinformation heightened fear, stress and anxiety levels (47). Anxiety stimulating fears of virus infectivity and spread simultaneously arose with the rapid and catastrophic emergence of the pandemic (45). Although medical students may have had some protective knowledge, they were not immune to the mental health burdens imposed on them as a result of the various implications of pandemic. Similar to the general population, medical students reported heightened levels of psychological distress, and academic disruption (48, 49).

### 4.3 Clinical vs. preclinical training phases

Three meta-anaylyses included in the review reported higher prevalences of depressive and/or anxiety symptoms for students in their preliminary years in medical school compared to those in their later years of study (26, 35, 38). One meta analysis conducted with 34,189 medical students in Africa; however, reported that second year medical students elicited a 76% decreased likelihood of developing depression compared to their counterparts (36). While most studies suggest higher anxiety and depression symptoms in early years, regional variations, curriculum or study design differences might account for the reported variations.

A meta analysis conducted by Puthran et al. (11) reported that Year 1 medical students had the highest rates of depression symptoms at 33.5%; however, this value gradually decreased to 20.5% by Year 5. Three cross-sectional studies (50–52) reported similar findings, with one reporting a 21.4% difference in symptoms of anxiety found in first-year students compared to sixth-year students. However, no significant difference was found between these groups for depressive symptoms. In contrast, a 2017 meta-analysis concluded a statistically insignificant difference regarding the prevalence of depression or depressive symptoms between preclinical students (23.7%) and clinical students (22.4%) (53). A similar report was found in a global meta-analysis that reported only a 0.2% difference in the prevalence of anxiety symptoms in clinical medical students compared to preclinical medical students, a result which was also deemed statistically insignificant. A cross-sectional study found depression to be more prevalent among fourth and fifth- year students as compared to second and third-year students. These inconsistencies may be attributed to methodological differences across studies, such as variations in screening tools, variations in the cut-off scores for the definitions of depressive symptoms, sample sizes, as well as regional and institutional factors.

Nonetheless, students transitioning from the preclinical years to their clinical years of study may feel anxious about the process as they are forced to identify gaps in their knowledge from basic sciences and are faced with a heavy workload (54). Students may also struggle with adapting to the new learning demands and critically applying the theoretical knowledge they attained during their basic science years in a practical manner (55). These factors may account for the existence of depression and anxiety symptoms among students in their clinical years; however, because they have already been exposed to the demanding nature of medical school in their preliminary years, they may have already attained the coping strategies and resilience needed to quickly adjust and integrate into their new environment. Preclinical students, on the other hand, have not been exposed to the unique and strenuous nature of medical school, and will likely spend their preliminary years developing new study habits and general routines. This may account for the increased prevalence of depressive and anxiety symptoms observed in preclinical students in the majority of studies.

### 4.4 The impact of continent of study on student mental health

Of the multiple meta-analyses that conducted subgroup analyses within their study, many reported significantly varied prevalences of depression and anxiety symptoms among medical students based on region, country, and continent. One meta-analysis by Jin et al. (31) reported a pooled prevalence of depression at 34% in North China and 18% in South China. This heterogeneity was attributed to the diversity of medical education in different regions in China (31). Similarly, Mekonnen et al. (36), reported significant variations in the prevalence of depression symptoms among medical students located within different countries across Africa (36). During the COVID-19 pandemic, one study reported a lower pooled prevalence of depression in Asian medical students compared with African, North or South American students. The study suggested that the difference was attributed to cultural differences regarding mental health across the countries. Specifically, the stigmatization of mental health in several Asian countries may result in inaccurate self-report measures of mental health disorders. As this study was conducted with data from the COVID-19 pandemic, it is also plausible that the locations that suffered the most impact from the pandemic consequently experienced greater increases in the prevalence of depression and anxiety symptoms. These suggestions are consistent with a study that declared that North America and Europe had the highest cases and deaths per capita, followed by South America, with Africa showing fewer deaths, and Asia reporting mixed impact (56). The meta-analysis by Jia et al. (33) supported these findings and reported a pooled prevalence of depression to be 52.3% in Europe and 36.2% in Asia. One systematic review (57) also reported mixed prevalences of both anxiety and depression symptoms across 47 different surveys conducted by medical students from multiple institutions across the globe.

Additional factors driving variability within continents may be attributed to economic variations, online learning limitations, and healthcare systems.

### 4.5 Biological sex differences in student mental health

A 2019 global meta-analysis conducted among medical students reported that females had a 38.0% prevalence of anxiety symptoms compared to their male counterparts, who had 27.6%; however, this relationship was termed statistically insignificant. Similar reports were found in studies by Dutta et al. (27) and Jin et al. (31) who determined the pooled prevalence of depressive symptoms to be 38 and 26% among females and 34 and 29% among males, respectively. An individual participant data meta-analysis found that female medical students exhibited significantly increased depression scores than their male counterparts (26). In contrast, a meta-analysis conducted among medical students in Africa determined that females were 75% less likely to develop depression. This is contrary to a common study hypothesis stating that mild to moderate likely depression is more likely to be reported by females than males (58). This discrepancy may be largely attributed to contextual factors such as symptom presentation, cultural norms, or specific stressors. Specifically, in sub-Saharan Africa, biological sex differences are evident in the manifestations and prevalence of depressive symptoms. Thus, the manifestations of depression are largely somatic, spiritual in nature, or based on interpersonal relationships, which are all factors that may obscure the detection of depression (59). Furthermore, female sex steroids or hormones have been implicated in the sex differences observed in certain psychiatric disorders (60). As a result of this, standardized screening tools may be unable to fully encompass the burden of disease, especially among women in these populations. The overall variation in the results regarding biological sex highlights the complex nature of biological sex related differences in mental health among medical students. Evidently, context-specific factors, such as cultural norms, stigma, and symptom expression, may have a substantial influence on the reporting and detection of mental health ilnesss.

## 5 Conclusions, implications and recommendations

The findings of this review have important implications for both the current and long-term mental wellbeing of medical students. Prominent reports of depression and anxiety symptoms in this study population suggest that psychological distress is a significant and prevalent concern. From a systems perspective, supporting the psychological health of medical students should be recognized as a strategic investment in workforce resilience. Failure to address these mental health challenges at their onset may positively contribute to an increased likelihood of reports of medical errors when students eventually become physicians (61). As preclinical students appear especially vulnerable, the implementation of early targeted interventions during the initial stages of training would be beneficial in mitigating subsequent mental health deterioration in students. The global institutionalization of targeted interventions in medical school systems would be a worthwhile investment in student mental health. A substantial body of evidence explores the effectiveness of multiple interventions on student mental health. Medical schools could implement multiple interventions curated to different student populations. A randomized control trial conducted by Wang et al. (62) observed a significant decrease in depression symptoms compared to the control group after the implementation of a targeted intervention program [internet-based, self-help, acceptance and commitment therapy program (iACT)] designed to reduce symptoms of depression, anxiety, and other forms of psychological stress among postgraduate Chinese medical students (62). Another study demonstrated that the implementation of counseling services among medical students served to reduce levels of anxiety and depression symptoms compared to those who did not receive counseling service (63). Additionally, cognitive behavioral therapy (CBT) based supportive text messages programs delivered via mobile or email such as Text4Mood, Text4Hope have been reported to be effective among the youth and general population in supporting their mental wellbeing and this can be adopted among medical students (64–66). Furthermore, the integration of mental health literacy programs and accessible, stigma-free counseling into medical curricula could serve to strengthen coping capacity.

Despite a growing body of literature, several gaps remain. Inconsistencies in study results were highly attributed to heterogeneity in screening instruments, definitions, and cut-off scores for standardized scales, which are all factors that further complicated cross-study comparisons. There was also a limitation in the number of studies that applied biological sex and culture-sensitive frameworks that account for alternative symptom presentations or the influence of cultural norms on self- reporting. An additional limitation existed with the exploration of factors such as socioeconomic status, ethnicity, or pre- existing mental health conditions, and their impact on mental health manifestations. Additionally, gender identity was poorly accounted for in the determination of risk factors for poor mental health outcomes of medical students. In general, there was a lack of data that recorded statistically significant correlates for depression and anxiety symptoms among medical students. This underscores the need for further empirical research to establish such correlates to strengthen the existing body of knowledge.

Addressing these gaps with additional longitudinal studies that follow medical students from admission into training would provide additional insights into the incidence and progression of mental health disorders. Greater attention should be paid to underrepresented regions and to developing culturally adapted interventions that consider local stigma and presentation patterns. The implementation of cost-effective mental health interventions should be promptly implemented into medical schools worldwide to ensure that each student has equal access to care. For policy and educational practice, medical schools should embed structured mental health support into their programs, explore the effectiveness of peer mentorship networks, and implement workload management strategies, particularly during times of crisis. By prioritizing these interventions, institutions and healthcare systems can better safeguard the mental wellbeing of our future physicians. Future systematic review may focus on the ethical aspects of mental health screening in medical students.

## 6 Limitations

Several limitations were identified within this scoping review that should be appropriately considered when interpreting the findings. Primarily, the search criteria were limited only to selected databases and English-language publications, which may have excluded potentially relevant studies published in other languages or identified elsewhere, like gray literature. Additionally, there is the probability of the same primary studies being included multiple times in the included systematic review and meta-analysis which may lead to the risk of double counting or duplication bias. Thus, consistent with the scoping review methodology, we did not evaluate the strength of included studies; therefore, the quality of the evidence underlying prevalence estimates and correlates cannot be assessed. Lastly, variations in study design, measurement tools, and definitions of anxiety and depression across studies limit direct comparisons and contribute to heterogeneity in reported prevalence rates.

## Data Availability

The original contributions presented in the study are included in the article/supplementary material, further inquiries can be directed to the corresponding author.
